# Ocular Circulation and Chronic Ocular Ischemic Syndrome before and after Carotid Artery Revascularization Surgery

**DOI:** 10.1155/2012/350475

**Published:** 2012-12-20

**Authors:** Shoichiro Kawaguchi, Jun-ichi Iida, Yoshitomo Uchiyama

**Affiliations:** Department of Neurosurgery, Nara Prefectural Nara Hospital, Nara 631-0846, Japan

## Abstract

*Background*. We evaluated the effect of carotid revascularization surgery on ocular circulation and chronic ocular ischemic syndrome (OIS). *Methods*. We examined ninety patients with carotid artery stenosis (more than 50% stenosis) at its origin treated with carotid endarterectomy (*N* = 56) or carotid artery stenting (*N* = 34). Twenty-five patients (28%) complained of chronic OIS. Ocular circulation was examined before and after revascularization surgery using ophthalmic artery (OphAr) and central retinal artery (CRA) color Doppler flow imaging. *Results*. (1) Ocular circulation: preoperatively, the average OphAr peak systolic flow velocity (Vs) was 0.05 m/sec, and the average CRA Vs was 0.07 m/sec. At 1 week after surgery, the average OphAr Vs significantly increased to 0.32 (*P* < 0.05), and the average CRA Vs significantly increased to 0.11 m/sec (*P* < 0.05). These significant improvements were sustained throughout the three months of the followup. (2) OIS: during the follow-up period (mean: 3.6 years), 15 patients (60%) showed visual acuity improvement, and no patients complained of amaurosis fugax or worsening of the chronic OIS. *Conclusion*. Carotid revascularization surgery was effective in improving the ocular circulation, and it was also useful for the chronic OIS due to the carotid artery stenosis.

## 1. Introduction

One of the important clinical aspects of internal carotid artery stenosis at its origin is the influence on the flow dynamics of the ocular circulation. The blood supply to the eye is mostly provided by branches of the ophthalmic artery (OphAr), which is a branch of the internal carotid artery. This is why many patients with cerebral ischemia in the internal carotid artery may present with ipsilateral visual symptoms [1]. The disturbed ocular circulation correlates with chronic ocular ischemic syndrome symptoms such as frequent amaurosis fugax or a decline of visual acuity [2, 3]. Carotid revascularization surgery, such as carotid endarterectomy (CEA) and carotid artery stenting (CAS), may also restore the cerebral perfusion pressure and improve the intracranial vascular hemodynamics including ocular circulation. Therefore, carotid artery revascularization surgery reduces the risk of stroke in symptomatic and asymptomatic patients [4, 5]. Despite this well-established benefit, there have been few reports concerning the effect of carotid revascularization surgery on ocular circulation. Therefore, it is significant to understand the ocular circulation and ocular symptoms in patients with internal carotid artery stenosis before and after CAS.

In this study, we discussed and analyzed the effect of carotid revascularization surgery on the ocular circulation and chronic ocular symptoms in patients with carotid artery stenosis.

## 2. Patients

From January 2002 through January 2012, we examined the ocular circulation in 90 consecutive patients showing internal carotid artery origin stenosis (more than 50%) treated with CEA or CAS. Eighty patients were males, and 10 were females. Their ages ranged from 47 to 81 years, with a mean of 69 years. The clinical symptoms of the patients were transient ischemic attack in 65 patients, stroke in 13, and asymptomatic in 12. 

According to the criteria of the NASCET study [4], the grades of angiographical internal carotid artery stenosis on the ipsilateral side were more than 60% stenosis in 10 patients, more than 70% stenosis in 17, more than 80% stenosis in 13, more than 90% stenosis in 34, and more than 95% stenosis in 16. Among the 90 patients, 25 complained of chronic ocular ischemic syndrome. All these 25 patients had visual acuity of 20/40 or worse, and 9 patients among them had frequent amaurosis fugax. No patients showed blindness. The exclusion criteria for chronic ocular ischemic syndrome were acute ocular ischemic symptoms such as sudden loss of vision, a single episode of amaurosis fugax, or ocular/orbital pain [6]. 

Carotid revascularization surgery was performed at least 4 weeks after the last attack. Within 2 weeks after the procedure, the patency of the treated carotid artery was confirmed by carotid angiography, MRA, or ultrasonography. No patients complained of a permanent neurological deficit due to the surgery.

All patients were followed up for the clinical symptoms after carotid revascularization. The follow-up period was 0.5 to 4.5 years (mean: 3.6 years). During this period, none of the patients had a recurrent ischemic attack or worsening of the symptoms including the ocular signs. 

## 3. Methods

To evaluate the ocular circulation, we examined the OphAr flow and central retinal artery (CRA) flow using color Doppler flow imaging (CDFI) with a 5-MHz phased linear probe; the mechanical index was less than 0.23, and the examination was completed within 3 minutes in each eye. 

**Figure 1:**
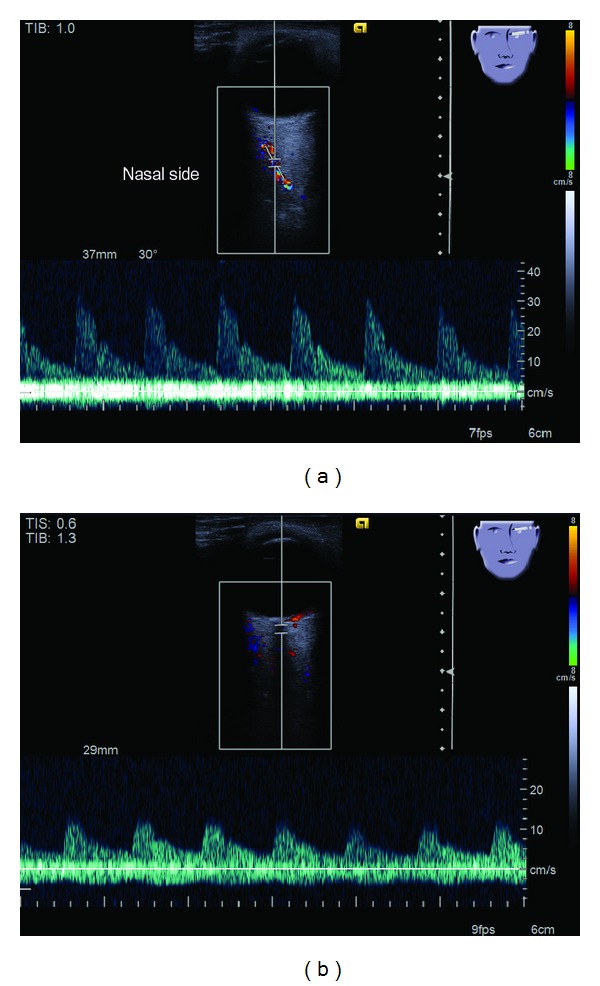
Color Doppler flow imaging of the ophthalmic artery (a) and central retinal artery (b) in normal control.

While the patient was supine, the probe was applied to the closed eyelids. During the examination, minimal pressure was applied to the globe to avoid the damage to the globe and artifacts. With the probe resting on the closed eyelids, the ultrasound beam was directed posteriorly in the orbit. After systematic scanning of the orbit, the OphAr and CRA were imaged, and spectral velocity analysis was performed. Pulsed Doppler spectrum analyses were recorded from the OphAr and CRA [7]. Blood flow was monitored continuously, and we employed a Doppler angle of 60° or less for each measurement [8]. Each Doppler waveform was automatically drawn and calculated using the software included with the ultrasound system. The OphAr was examined approximately 20 mm behind the globe as the vessel parallel to the nasal border of the optic nerve just after crossing it, and the CRA was examined within 5 mm of the retrolaminar portion of the optic nerve (Figure 1) [8]. The OphAr and CRA CDFI findings from the ipsilateral side of revascularization surgery were analyzed. The OphAr and CRA CDFI were performed within 1 week before surgery and at 1 week, 1 month, and 3 months after surgery in each patient. These CDFI studies provided information regarding the flow direction and peak systolic flow velocity of the OphAr and CRA.

We also performed OphAr and CRA CDFI on 51 normal healthy volunteers. Their mean age was 68 years; 28 volunteers were males, and 23 were females. The average peak systolic flow velocities were 0.35 ± 0.13 m/sec in the OphAr and 0.12 ± 0.04 m/sec in the CRA. All volunteers showed normal OphAr flow direction, that is, flow away from the orbital apex to the globe. 

All patients were examined in terms of their ocular symptoms such as visual acuity and amaurosis fugax before the CEA or CAS procedure and during the follow-up period. 

The physiological data were compared using two-tailed paired Student's *t*-test and chi-square test. A value of *P* < 0.05 was considered as the threshold for statistical significance. All values are reported as mean ± standard deviation (SD). 

## 4. Results

### 4.1. Ocular Circulation (Table 1)

#### 4.1.1. Before Surgery

The OphAr flow directions were reversed in 25 patients and antegrade in 65 patients. The average OphAr peak systolic flow velocity was 0.05 ± 0.34 m/sec, and the average CRA peak systolic flow velocity was 0.08 ± 0.02 m/sec. These values were significantly low compared with those of the controls (*P* < 0.05). 

There was a statistically significant difference (*P* < 0.05) between the values of the average CRA peak systolic flow velocity in the patients with reversed OphAr flow, which was 0.06 ± 0.01 m/sec, and antegrade OphAr flow, which was 0.08 ± 0.02 m/sec. 

#### 4.1.2. After Surgery

At 1 week after surgery, the patients showing reversed OphAr flow before surgery all returned to the normal antegrade OphAr flow. This correction of the reversed OphAr flow was significant (*P* < 0.05). At 1 month and 3 months after surgery, the significant correction of the ophthalmic artery flow was sustained. At one week after surgery, the average OphAr peak systolic flow velocity significantly increased to 0.32 ± 0.12 m/sec (*P* < 0.05), and the average CRA peak systolic flow velocity also significantly increased to 0.11 ± 0.03 m/sec (*P* < 0.05). These significant increases of the average OphAr and CRA peak systolic flow velocities were sustained throughout the three months of the followup.

### 4.2. Ocular Symptoms

Before surgery, 25 patients complained of chronic ocular ischemic syndrome. Among these 25 patients, 15 showed reversed OphAr flow direction, and the other 10 patients showed antegrade flow. The relationship between the presence of chronic ocular ischemic syndrome and reversed OphAr flow direction was significant (*P* < 0.05). In addition, the average OphAr and CRA peak systolic flow velocities were significantly low (*P* < 0.05) in the patients with chronic ocular ischemic syndrome compared with those in the patients without chronic ocular ischemic syndrome (Table 2). Among 25 patients with chronic ocular ischemic syndrome before surgery, 15 patients (60%) showed improvement of visual acuity during the follow-up period, but 10 patients had no improvement of visual acuity, even with normal ophthalmic flow after surgery. This failure might have been due to irreversible neovascular glaucoma. In each patient, amaurosis fugax was not seen during the follow-up period. 

Sixty-five patients showed no chronic ocular ischemic syndrome before surgery. During the follow-up period, these 65 patients complained of no chronic ocular ischemic syndrome, such as disturbance of visual acuity and frequent amaurosis fugax. 

### 4.3. Illustrative Case

**Figure 2:**
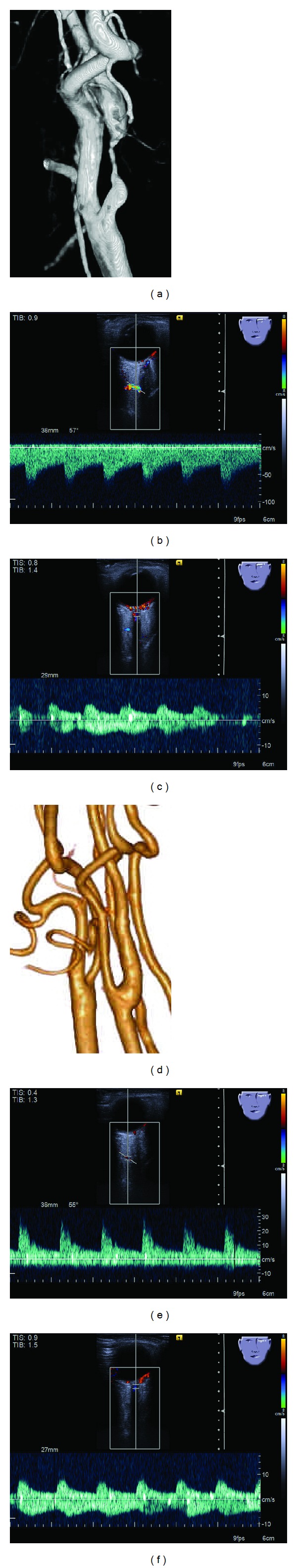
Left carotid angiography (a), ophthalmic artery Doppler flow imaging (b), and central retinal artery Doppler flow imaging (c) before carotid endarterectomy were shown. 3D CT angiography (d), ophthalmic artery Doppler flow imaging (e), and central retinal artery Doppler flow imaging (f) after carotid endarterectomy were shown.

A 77-year-old man was referred to our hospital complaining of transient right hemiparesis and left visual acuity decline. Left carotid angiography showed 98% stenosis of the right internal carotid artery at its origin (Figure 2(a)). The left OphAr CDFI showed reversed flow, and the peak systolic flow velocity was −0.62 m/sec (Figure 2(b)). The left CRA peak systolic flow velocity was 0.06 m/sec (Figure 2(c)). Left CEA was performed. Postoperative carotid angiography showed the good patency of the carotid artery (Figure 2(d)). At 1 week after CEA, the left OphAr CDFI showed resolution of the reversed flow, and the peak flow velocity was 0.30 m/sec (Figure 2(e)). The peak systolic flow velocity of the CRA was 0.12 m/sec (Figure 2(f)). At 1 month and 3 months after CEA, there was no marked change of the peak systolic flow velocity of OphAr and CRA. 

The right visual acuity gradually improved at 3 months after CEA. The patient was followed up for 2.8 years after CEA, and there were no neurological ischemic events including visual symptoms.

## 5. Discussion

Patients with internal carotid artery occlusion or stenosis develop ocular ischemia because these diseases reveal the hemodynamic reduction of ocular circulation [9]. The presentation of ocular signs can vary considerably may be quite varied, with some cases showing rapid advancement of neovascularization following high intraocular pressure [10, 11]. Neovascular glaucoma secondary to internal carotid artery occlusion is usually resistant to treatment [11]. Moreover, carotid artery occlusion often progresses without symptoms, and when the patient notices an ocular disorder and visits a clinic, the condition is often at an advanced stage of ocular ischemia, in which neovascular glaucoma has developed with severe internal carotid artery stenosis [11]. Therefore, it is significant to evaluate the ocular circulation to detect and prevent ocular ischemia in patients with internal carotid artery stenosis treated with a CEA or CAS procedure. We previously reported the effect of superficial temporal artery to middle cerebral artery (STA-MCA) bypass, CEA, and CAS on the OphAr flow [12–14]. However, there have been few reports about the effect of carotid revascularization surgery on the ocular circulation and ocular ischemic syndrome. In this study, we evaluated the effect of carotid revascularization surgery, such as CEA and CAS, on chronic ocular ischemic syndrome and ocular circulation, including the OphAr flow and CRA flow, before and after surgery as well as during the follow-up period. 

Reversed OphAr flow seen in the patients with carotid artery stenosis may contribute to the development of ocular ischemic syndrome [2]. In these patients, the ophthalmic artery might function as collateral circulation from the extracranial to the intracranial circulation [15]. When severe internal carotid artery stenosis occurs in patients with incomplete blood circulation in the circle of Willis, the blood flow in the OphAr reverses to supply the ipsilateral brain. This so-called “steal phenomenon” results in ocular ischemia, the prolongation of which leads to rubeosis iridis and neovascular glaucoma [9]. In this series, 25 patients showed reversed OphAr flow before CEA or CAS, and the reversed ophthalmic artery flow was significantly seen in the 15 patients (60%) showing chronic ocular ischemic syndrome before revascularization surgery in this series. 

Yamamoto et al. established four eye types based on the retrobulbar blood flow direction [9]: forward OA, CRA, and short posterior ciliary artery (SPCA) flow (type 1); reverse OA and forward CRA and SPCA flow (type 2a); reverse OA and undetectable CRA and SPCA flow (type 2b); and undetectable flow in all three arteries (type 3). In type 2b eyes, almost the entire collateral circulation via the OphAr was deviated towards the internal carotid artery, reducing the blood flow to the eye. Rubeosis iridis occurred in half of them. In type 3, no collateral blood supply developed. Therefore, the ocular end circulation was markedly reduced, possibly because of insufficient collateral circulation via both the circle of Willis and the external carotid artery system. Rubeosis iridis was seen in one-third. In our series, 15 patients with reversed OphAr flow showed ocular ischemic syndrome, and those patients also showed the significantly reduced CRA flow. Therefore, eyes with reversed OphAr flow and low CRA peak systolic flow velocity may be at greater risk for ocular ischemic syndrome. 

Carotid artery revascularization reduces the risk of stroke in symptomatic and asymptomatic patients [5]. Improvements in surgical and endovascular techniques have reduced the incidence of ischemic stroke following CEA and CAS, respectively. In this series, after the carotid revascularization surgery, the average OphAr and CRA peak systolic flow velocities improved to the normal control levels. Moreover, after the carotid revascularization surgery, the flow direction of the OphAr became normal in all cases showing reversed OphAr flow before surgery. This significant improvement in the ophthalmic artery flow velocity also explains the correction of ocular hemodynamic compromise. 

In this study, improvement in the peak systolic flow velocity of the OphAr and CRA flows and the normalization of the reversed OphAr flow occurred 1 week after revascularization surgery. The significant improvements of the average OphAr and CRA peak systolic flow velocities and the correction of the ophthalmic artery flow direction were sustained throughout the three months of followup. These effects of carotid revascularization surgery were highly expected. We clarified the chronological improvement of the disturbed OphAr and CRA peak systolic flow velocities and the correction of the reversed OphAr flow direction after revascularization surgery using the OphAr and CRA CDFI findings. The OphAr and CRA CDFI findings provide clear evidence of hemodynamic compromise in carotid artery stenosis. We proved the significantly increased flow velocity and corrected flow direction of the ocular circulation in all patients immediately after CEA and CAS. 

For the patients with chronic ocular ischemic syndrome due to disturbed ocular circulation, it is vital to correct the ocular circulation to prevent and improve ocular ischemia [15]. In this series, 25 patients showed chronic ocular ischemic syndrome. Among them, 15 patients showed reversed ophthalmic artery flow initially. After CEA or CAS, the reversed ophthalmic artery flow in all of these 15 patients was resolved. Therefore, revascularization surgery is the appropriate treatment maneuver for patients with ocular ischemic syndrome due to reversed ophthalmic artery flow revealed from severe internal carotid artery stenosis. The other 10 patients among those with chronic ocular ischemic syndrome showed antegrade ophthalmic artery flow with reduction of peak systolic flow velocity before CAS or CEA. In these patients, OphAr and CRA peak systolic flow velocities significantly increased immediately after CAS procedure. Fifteen patients showed improvement of the visual symptoms after surgery. Ten patients showed no improvement of the chronic ocular ischemic syndrome due to irreversible optic apparatus lesion. 

We report clear evidence of the effect of CEA and CAS on the improvement and prevention of chronic ocular ischemia due to internal carotid artery stenosis, on the basis of data obtained from OphAr and CRA CDFIs and clinical symptoms. Therefore, there is a good correlation between the course of ocular ischemic syndrome and the improvement of the ophthalmic artery CDFI findings during the follow-up period. CEA and CAS are effective for the treatment and prevention of ocular ischemic syndrome and are most beneficial if performed early, before the onset of irreversible neovascular glaucoma or irreversible ischemic optic fundi [10].

Since Lieb et al. [16] first reported CDFI as a reliable means of evaluating ocular circulation, and it has been used to measure retrobulbar blood flow in occlusive carotid artery disease patients and to confirm the presence of the steal phenomenon [9]. CDFI of the ocular circulation is a noninvasive, repeatable technique for measuring orbital blood flow. The hemodynamic parameters of CDFI are not sex dependent and do not vary between orbits [9]. The OphAr is easily visualized deep in the orbital cavity, in the area where it crosses the optic nerve; the spectral analysis is typical, displaying a pulsatile and positive waveform with blood flow velocity of 35 ± 11 cm/sec [17]. In the present series, OphAr and CRA could also be detected using orbital color Doppler flow imaging in all patients and normal volunteers. Reliability problems can arise when blood flow monitoring is performed in small ocular arteries or in veins because the lower velocities found in these vessels are close to the resolution limit for detection using the ultrasonographic system (when the flow rate, e.g., falls below 1–3 cm/sec in central retinal vein occlusion) [17]. Ultrasonographic waves can damage the eye tissues, particularly from heat; therefore, the avoidance of prolonged imaging is recommended, as is the reduction of output energy [18]. In our series, the MI was less than 0.23, and examination time was less than 3 minutes. 

## 6. Conclusion

In patients with carotid artery stenosis, reversed OphAr flow is related to the decrease of CRA peak systolic flow velocity and the occurrence of chronic ocular ischemic syndrome. Carotid revascularization surgery achieved normalization of the disturbed OphAr flow and CRA flow, whether the flow direction of the OphAr was reversed or antegrade, immediately after revascularization surgery. CEA and CAS improved the chronic ocular ischemic syndrome revealed from severe carotid artery stenosis and also prevented the progression and onset of chronic ocular ischemic syndrome.

## Figures and Tables

**Table 1:** Course of peak systolic flow velocity (m/sec).

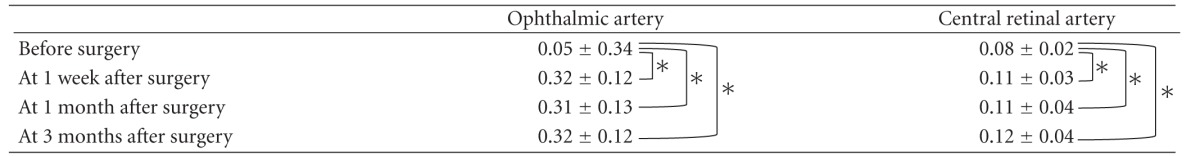

*P < 0.05.

**Table 2:** Relationship between chronic ocular ischemic syndrome and ocular circulation.

	Chronic ocular ischemic syndrome		
	Positive		Negative
Peak systolic flow velocity (m/sec)			
Ophthalmic artery	−0.19 ± 0.4	(*P* < 0.05)	0.14 ± 0.26
Central retinal artery	0.06 ± 0.01	(*P* < 0.05)	0.08 ± 0.02
Ophthalmic artery flow direction (cases)			
Antegrade	10	(*P* < 0.05)	55
Reversed	15		10
